# Indecisiveness moderates the relationship between rumination modes and depressive symptoms

**DOI:** 10.3389/fpsyg.2025.1681121

**Published:** 2025-11-06

**Authors:** Brandon Winchell, Iony D. Ezawa

**Affiliations:** Department of Psychology, University of Southern California, Los Angeles, CA, United States

**Keywords:** rumination, indecisiveness, depression, cognition, moderation, decision-making

## Abstract

**Introduction:**

Major depressive disorder is a debilitating and common mental health condition. Rumination and indecisiveness are both well-established cognitive risk factors for depressive symptoms, but their interactive effects remain underexplored. Drawing on theories about rumination's level of construal, which distinguish between abstract and concrete modes of thinking, this study examined whether aversive indecisiveness moderates the relationships between rumination modes (abstract and concrete) and concurrent depressive symptoms.

**Method:**

We recruited two samples: an undergraduate student sample (Sample 1, *N* = 412) and a general population sample (Sample 2, *N* = 258). Participants completed self-report measures of depressive symptoms, rumination modes, and indecisiveness. Robust linear regression was used to test moderating effect of aversive indecisiveness on rumination modes while controlling for gender, age, income, and timepoint.

**Results:**

Aversive indecisiveness significantly moderated the relationship between abstract rumination and depressive symptoms in both samples, such that the positive association was stronger at higher levels of indecisiveness. In Sample 2, aversive indecisiveness also moderated the relationship between concrete rumination and depressive symptoms, such that the negative association was stronger at higher levels of indecisiveness.

**Discussion:**

These results suggest that the co-occurrence of abstract rumination and aversive indecisiveness may confer heightened risk for depressive symptoms, highlighting the importance of considering both factors jointly in understanding and treating depression.

## 1 Introduction

Major depressive disorder (MDD) is a leading cause of poor health and disability worldwide (World Health Organization, 2017, 2023). Although MDD is a heterogeneous disorder that can present idiosyncratically, one common feature is the cyclical and pervasive nature of negative, inaccurate thought patterns characterized by self-criticism and self-defeating beliefs (Beck, 1961, 2019). A key challenge for many with MDD is difficulty disengaging from such negative thoughts, which often takes the form of rumination.

### 1.1 Rumination

Rumination is a cognitive emotion regulation strategy that involves repetitively and passively focusing on distress, its causes, and consequences, without leading to active problem solving (Nolen-Hoeksema et al., 2008). Since its initial description in (Nolen-Hoeksema 1991) response styles theory, rumination has gained evidence as a well-established risk factor for depression with meta-analyses showing a medium to large association between rumination and depressive symptoms (Aldao et al., 2010; Rood et al., 2009). Moreover, rumination has been found to be a transdiagnostic risk factor for anxiety and eating disorders and self-injurious behavior (McLaughlin and Nolen-Hoeksema, 2011; Nolen-Hoeksema, 2000; Nolen-Hoeksema et al., 2008). Experimental studies have also provided evidence of rumination's causal role in worsening depressive processes. For example, (Nolen-Hoeksema and Morrow 1993) found that inducing rumination in depressed participants worsened mood, whereas distraction improved it. These findings have been replicated among clinical samples (Kuehner et al., 2023; Watkins and Brown, 2002) and with nonclinical samples in negative mood induction studies (Huffziger and Kuehner, 2009; Kuehner et al., 2009).

There are several proposed explanations for why rumination may be harmful. (Nolen-Hoeksema et al. 2008) theorized that rumination sustains negative emotional focus, impairs problem-solving, inhibits goal-directed behavior, and reduces social support. The negative effects of rumination may be intensified in individuals who also possess other risk factors like indecisiveness. For instance, someone who has difficulty making decisions due to rumination and other factors (e.g., anxiety about decisions) may face greater risk of depression than someone affected by only one of these vulnerabilities.

Although rumination is generally associated with negative outcomes, it may not always be maladaptive. Watkins (2008) reviewed forms of repetitive thought, which encompasses rumination. Watkins suggested that repetitive thought can be maladaptive due to its association with vulnerability for depression and anxiety as well as physical health impairments. Alternatively, repetitive thought can also be constructive, aiding recovery from traumatic events and depression, facilitating planning, and promoting healthy behaviors. To elucidate why repetitive thought can be both constructive and unconstructive, Watkins highlighted the level of construal of repetitive thoughts, or how abstract or concrete repetitive thoughts are.

Concrete thinking tends to be defined, clear, and focused on a particular situation, while abstract thinking is more vague, generalized, and difficult to pin down across contexts (Stöber and Borkovec, 2002). The reduced concreteness theory, originally applied to pathological worry by (Stöber and Borkovec 2002) and later applied to all repetitive thoughts by Watkins (2008), posits that an abstract mode of thinking may be maladaptive in difficult or emotionally distressing states whereas a concrete mode of thinking tends to be adaptive in these same states. Watkins and Moulds (2005) tested this hypothesis by randomly inducing either abstract or concrete rumination in depressed participants before administering social problem-solving vignettes. They found that while both modes of rumination led to negative mood and self-focus, concrete rumination (compared to abstract rumination) resulted in significantly more problem solving (measured by proposed solutions and more effective solutions as rated by judges). These findings suggest that the mode, or level of construal, of rumination may affect its consequences.

Accordingly, Watkins (2008) proposed the level-of-construal dysregulation hypothesis which posits that depression-prone individuals tend to have overly abstract modes of rumination, especially in difficult emotional states, where a concrete processing style would be more adaptive. Several studies substantiate that abstract rumination is positively associated with depressive symptoms (Di Schiena et al., 2013; Douilliez et al., 2014; Kambara et al., 2019; Ros et al., 2024). Evidence regarding the association between concrete rumination and depressive symptoms is mixed, with some studies supporting a negative association (Di Schiena et al., 2013; Douilliez et al., 2014) and others finding no significant association or one of minimal effect size (Kambara et al., 2019; Ros et al., 2024). Importantly, none of these studies suggested the existence of a meaningful positive association between concrete rumination and depressive symptoms.

This nuanced understanding of the role of rumination in depression is a step in the right direction. However, MDD is a heterogenous disorder and rumination mode alone does not fully account for individual differences in vulnerability to depression. Therefore, understanding how distinct but overlapping risk factors may interact with rumination mode could help illuminate for whom rumination is especially problematic. Watkins and Roberts (2020) recently proposed an integrated model that suggests that the emotional impact of rumination depends not solely on its mode (abstract vs. concrete), but also on moderating factors that influence whether rumination is hard to “break free from” or can be more easily regulated. Few studies have directly examined moderating factors directly, especially in the realm of decision-making and goal-directed behavior. One plausible and empirically relevant trait is indecisiveness.

### 1.2 Indecisiveness

Indecisiveness is broadly characterized as a tendency to find decision-making difficult and stressful (Germeijs and De Boeck, 2002). While momentary indecision is a common situation- and context-specific state, trait indecisiveness reflects a stable cognitive style that persists across situations and contexts (Frost and Shows, 1993; Germeijs and De Boeck, 2002; Rassin et al., 2007). This trait has been linked to a variety of psychopathology outcomes, including self-reported symptoms of obsessive-compulsive disorder, perfectionism, procrastination, and hoarding. Further work has shown indecisiveness is associated with other forms of psychopathology including depression and anxiety (Di Schiena et al., 2013; Hallenbeck, 2020; Rassin et al., 2007; Lauderdale et al., 2024; Lauderdale and Oakes, 2021). Its clinical relevance to depression is also underscored by the diagnostic criteria for MDD which indicates that individuals with depression can display a “diminished ability to think or concentrate, or indecisiveness” (American Psychiatric Association, 2013).

Aversive indecisiveness, which comprises the tendency toward possessing a decision-making process laden with negative affect and threat-oriented cognition, is a facet of indecisiveness that has been found to be predictive of intolerance of uncertainty, avoidance, anxiety symptoms, self-reported difficulty with attentional control, and emotional distress (Lauderdale and Oakes, 2021). This is in alignment with earlier findings about aversive indecisiveness (Spunt et al., 2009; Lauderdale et al., 2019) and suggests its importance as an especially critical risk factor for psychopathology.

### 1.3 Rumination-indecisiveness associations

Past work substantiates an association between trait level rumination and broadly defined indecisiveness, with a medium to large effect (Kožuchová and Jurásová, 2025; Di Schiena et al., 2013; Piotrowski, 2019). Other work has examined rumination alongside situational indecision, which can offer helpful insight into rumination's association with decision-making problems. For instance, in a study where college students were asked to devise plans to improve either their universities' housing policies or curriculums, those who scored high on measures of rumination tended, to be less confident, less committed, and express need for more time to formulate their plans compared to those who scored low on rumination measures (Ward et al., 2003). In other words, those with a tendency to ruminate showed greater indecision. Similarly, in an experimental task, van Randenborgh et al. (2010) found that induced rumination led dysphoric participants to rate decisions as more difficult and to feel less confident in their decisions as compared to participants in a distraction control. The relationship between group (i.e., rumination vs. distraction) and decision-making difficulty was fully mediated by presence of ruminative thought content, while the relationship between group and decision-making confidence was partially mediated by negative affect. Such findings substantiate rumination's role in leading to indecision due to both cognitive and emotional pathways.

To our knowledge, only one study has examined the link between abstract vs. concrete modes of rumination and indecision. Utilizing the same paradigm as van Randenborgh et al. (2010), Di Schiena et al. (2013) induced either abstract or concrete rumination and found that abstract rumination increased decision latency and perceived difficulty in making decisions, but only in individuals with elevated depressive symptoms. Their correlational findings also supported positive associations among abstract rumination, indecisiveness, and depressive symptoms. Conversely, concrete rumination was negatively associated with these same measures. Taken together, these findings suggest that abstract rumination may play a specific role in fostering decision-making difficulties and related depressive symptoms.

To date, no studies have directly examined indecisiveness, particularly aversive indecisiveness, as a moderator of the relationship between rumination modes and depressive symptoms. Although related, rumination and indecisiveness are dissociable constructs that may uniquely and interactively contribute to depression. Shared features between indecisiveness and abstract rumination (such as prolonged evaluation, generalized concerns, and difficulty initiating goal-directed behavior; Nolen-Hoeksema et al., 2008; Rassin et al., 2007) suggest that indecisiveness may amplify the depressive effects of abstract rumination. In contrast, concrete rumination, which focuses on specific situations and facilitates action, may help highly indecisive individuals make decisions and reduce symptoms.

### 1.4 Current study

The present study aimed to explore how rumination's two levels of construal (abstract vs. concrete) interact with aversive indecisiveness in predicting depressive symptoms. Building on Watkins' (2008) level-of-construal dysregulation theory and prior findings on decision-making impairments, we had two hypotheses. First, we predicted that aversive indecisiveness would moderate the relationship between abstract rumination and depressive symptoms, specifically that their positive relationship would be stronger among individuals with higher aversive indecisiveness. Second, we predicted that aversive indecisiveness would moderate the relationship between concrete rumination and depressive symptoms, specifically that their negative relationship would be weaker among individuals with higher aversive indecisiveness.

## 2 Methods

### 2.1 Participants

Two samples were collected for this study. The first sample (Sample 1) was comprised of college students at an institution in the U.S. The second sample (Sample 2) was a sample of general adults in the U.S. Both samples had the same inclusion criteria, namely that the participants endorsed (1) being fluent in English, (2) located in the U.S., and (3) being 18 years or older. Below we go into more detail about each sample.

#### 2.1.1 Sample 1

Undergraduate students were recruited from the subject pool at a large West Coast university and compensated with course credit. The sample was collected at two timepoints: August to September 2024 (*n* = 293) and January to March 2025 (*n* = 204). Participants from both timepoints shared similar values for key study variables, and therefore the samples were collapsed for the analyses (see Supplementary material for more details).

From the original total of 497 participants who completed the study, we removed 20 participants who completed the survey in quicker than half of the median completion time (the median was seven mins and 48 secs for the collapsed sample) and 64 participants who inaccurately answered any attention check item. This data quality approach is recommended to remove likely inattentive or low effort responses, which is common in online survey research (Hillygus and LaChapelle, 2022). Further, one participant was removed because they reported being 40 years old (greater than 12 SD above the mean reported age), which is not representative of college students. This process resulted in a final sample size of 412 participants. The majority (73.5%) of the sample identified as women and 26.5% as men. Regarding race and ethnicity, 34.5% identified as Asian, 26.5% as White, 10.4% as Hispanic, Latino, or Spanish origin, 4.1% as Black or African American, 3.4% as Middle Eastern/North African, 0.2% as American Indian/Alaska Native, 0.2% as Native Hawaiian/Pacific Islander and 20.4% as multiracial. The mean age was 20.0 years old (*SD* = 1.2 years). Full demographic information for the sample is displayed in Table 1.

**Table 1:** Participant characteristics for sample 1 and 2.

**Characteristics**	**Sample 1**		**Sample 2**	
	***n***	**%**	***n***	***%***
**Gender**				
Women	303	73.5	123	47.7
Men	109	26.5	125	48.4
Non-binary	0	0	6	2.3
Not listed, please specify	0	0	3	1.2
Prefer not to answer	0	0	1	0.4
Age, *M* (*SD*)	20.0	(1.2)	35.6	(12.2)
**Racial background**				
Asian	142	34.5	15	5.8
White	109	26.5	167	64.7
Hispanic, Latino, or Spanish Origin	43	10.4	17	6.6
Black or African American	17	4.1	32	12.4
Middle Eastern/North African	14	3.4	1	0.4
American Indian/Alaska Native	1	0.2	2	0.8
Native Hawaiian/Pacific Islander	1	0.2	0	0
Two or more races	84	20.4	22	8.5
Prefer not to answer	1	0.2	2	0.8
**Education**				
Did not complete high school	–^a^	–	3	1.2
High school	–	–	36	14.0
Part college	–	–	69	26.7
Two-year college	–	–	22	8.5
Four-year college	–	–	88	34.1
Graduate school, part or completed	–	–	37	14.3
Not listed, please specify	–	–	3	1.2
**Income**				
Less than $25,000	101	24.5	45	17.4
$25,000 to $34,999	11	2.7	25	9.7
$35,000 to $49,999	10	2.4	31	12.0
$50,000 to $74,999	18	4.4	54	20.9
$75,000 to $99,999	23	5.6	31	12.0
$100,000 to $149,999	27	6.6	44	17.1
$150,000 or more	88	21.4	19	7.4
Prefer not to answer	134	32.5	9	3.5

a Due to the nature of the subject pool, it can be assumed that all participants in Sample 1 were undergraduate students.

#### 2.1.2 Sample 2

Adults were recruited from the Prolific research database and compensated at a rate of approximately $18/hour. This sample was collected in July-August 2024 (*n* = 77) and February 2025 (*n* = 205). Participants recruited at both time points shared similar values for key study variables, and therefore the samples were collapsed for analyses (see Supplementary material for more details).

Following the same procedures used for Sample 1, we removed 16 participants who completed the survey quicker than in half of the median completion time (the median was seven mins and 50 secs for the collapsed sample) and 8 participants who inaccurately answered any attention check item. This resulted in a final sample size of 258 participants. Approximately half (47.7%) of the sample identified as women, 48.4% as men, and 2.3% as non-binary. Regarding race and ethnicity, 64.7% identified as White, 12.4% as Black or African American, 6.6% as Hispanic, Latino, or Spanish origin, 5.8% identified as Asian, 0.8% as American Indian/Alaska Native, 0.4% as Middle Eastern/North African, and 8.5% as multiracial. The mean age was 35.6 years old (*SD* = 12.2 years). Full demographic information of the sample is displayed in Table 1.

### 2.2 Measures

#### 2.2.1 Demographic questionnaire

Demographic information was gathered via a self-report questionnaire. The following data were collected: age, gender identity, sexual orientation, racial/ethnic background, educational attainment, employment status, household income, relationship status.

#### 2.2.2 Depressive symptoms

Depressive symptoms were assessed using the second edition of the Beck Depression Inventory (BDI-II; Beck et al., 1996). This is a well-validated and widely used 21-item measure for depressive symptoms. Its items reflect symptoms outlined by the fourth edition of the Diagnostic and Statistical Manual of Mental Disorders (DSM-IV; American Psychiatric Association, 1994), which are highly consistent with the DSM-5 (Substance Abuse Mental Health Services Administration, 2016, p. 24–25). Each item is rated on a scale from 0 to 3, with total scores ranging from 0 to 63; higher scores reflect greater symptom severity. The single item assessing suicidality was removed for this study; therefore, we used prorated total scores. A review article assessing the psychometric properties of the BDI-II shows that it has demonstrated high internal consistency (α = 0.90), high test-retest reliability (coefficients from 0.73 to 0.96), and high concurrent validity with other depressive symptom scales (coefficients from 0.66 to 0.86; Wang and Gorenstein, 2013). In the current sample, we also observed high levels of internal consistency (α = 0.92, 95% confidence interval (CI) [0.91, 0.93] in Sample 1, α = 0.95, 95% CI [0.94, 0.96] in Sample 2).

#### 2.2.3 Rumination

Abstract and concrete rumination were assessed using the Mini Cambridge-Exeter Repetitive Thought Scale (Mini-CERTS; shortened version created in French by Douilliez et al., 2014 [with both English and French translations included] adapted from the longer, English version developed by Barnard et al., 2007). The Mini-CERTS is a 16-item questionnaire where each item is rated on a 4-point scale (1 = almost never, 4 = almost always), preceded by the following prompt: “When thoughts, feelings, and situations or events about me come to mind...” Items such as “My thinking tends to become open, loose, expansive, and creative” attempt to capture abstract rumination. Alternatively, items such as “I seem to be engaged and in touch with what is going on around me” attempt to capture concrete rumination.

Research from Douilliez et al. (2014) supports Mini-CERTS' psychometric properties. It has a conceptually driven two-factor model with factors representing abstract and concrete modes of thinking/ruminating. Sum scores for each subscale are calculated with higher scores reflecting greater abstract or concrete modes of rumination. Previous use indicates acceptable to good internal consistency for both subscales (α = 0.71 to 0.80; Di Schiena et al., 2013; Douilliez et al., 2014).

Because little previous work has been done with the Mini-CERTS in English, we conducted an exploratory factor analysis (EFA) to examine the factor structure of this measure in our samples. For a detailed account of this process, see the Supplementary material. All items exhibited loadings of λ ≥ 0.3 on factors corresponding to their theorized subscales with no substantial cross-loadings. The only exception, found in Sample 1, was Item 16, which possessed an equally large loading on both factors. Therefore, Item 16 was excluded from the calculation of subscale scores in both samples.^1^ The abstract rumination subscale showed good internal consistency (α = 0.81, 95% CI [0.78, 0.84] in Sample 1, α = 0.85, 95% CI [0.83, 0.88] in Sample 2). The concrete rumination subscale showed acceptable internal consistency (α = 0.71, 95% CI [0.67, 0.75] in Sample 1, α = 0.79, 95% CI [0.74, 0.83] in Sample 2).

#### 2.2.4 Aversive Indecisiveness

To measure indecisiveness, we utilized the aversive indecisiveness subscale (identified by Lauderdale and Oakes, 2021) of Revised Indecisiveness Scale (RIS; Rassin et al., 2007). The RIS is an eleven-item scale focused on the affective, cognitive, and behavioral dimensions of indecisiveness. Items are rated on a 5-point scale (1 = strongly disagree, 5 = strongly agree). The aversive indecisiveness subscale covers four items and therefore subscale scores range from 4 to 20, with higher scores indicating more aversive indecisiveness. The RIS has demonstrated good internal consistency (α = 0.86), high test-retest reliability (*r* = 0.88) and was significantly correlated with psychopathology and conceptually related measures, suggesting high convergent validity (Rassin et al., 2007). Lauderdale and Oakes (2021) detail a theoretically motivated factor structure separating the RIS into two subscales, one related to positive attitudes and the other related to aversive indecisiveness. Utilizing their structure, we conducted a CFA. The fit of this CFA was good in both samples (CFI's ≥ 0.95, TLI's ≥ 0.97, RMSEA's ≤ 0.08, SRMR's ≤ 0.045). Also, items for the aversive indecisiveness factor demonstrated strong standardized loadings (λ ≥ 0.65 in Sample 1, λ ≥ 0.73 in Sample 2; see Supplementary material for all estimates). The items of this factor were then used to create a sum score. In our two samples, this sum score demonstrated good internal consistency (α = 0.80, 95% CI [0.77, 0.84] in Sample 1, α = 0.86, 95% CI [0.83, 0.89] in Sample 2).

#### 2.2.5 Attention check items

Four questions were included in the survey to check that participants were paying attention and not answering in a random or pre-determined manner. The content of these items is unrelated to the other questionnaires, and they were formulated to be clear to those carefully reading the questions. An example of one of these questions is “What day is typically considered part of the weekend?” with four options, “Saturday” being the correct answer.

### 2.3 Procedure

The study was administered at a single timepoint via an online Qualtrics survey. Participants completed questionnaires to assess their demographics, depressive symptoms, trait levels of rumination (i.e., abstract and concrete), and indecisiveness. The order of the questionnaires and attention-check items was randomized between participants to reduce potential order effects. All study procedures were reviewed and approved by our institutional review board.

### 2.4 Data analysis overview

#### 2.4.1 Preliminary analysis

Descriptive statistics and correlations were computed for key study variables (depressive symptoms, indecisiveness, and rumination modes). We used Welch's independent samples *t*-tests to investigate potential gender differences between men and women for key study variables.

#### 2.4.2 Primary analysis

To test our primary hypotheses, we used multiple regression analysis to test the interaction effect of rumination modes and aversive indecisiveness on depressive symptoms. We also controlled for gender, age, income, and timepoint in both Samples. Gender and timepoint were contrast-coded such that woman, timepoint 1 = − 0.5 and man, timepoint 2 = 0.5. The addition of the demographic covariates was motivated by the literature regarding gender and age differences between key study variables (Girgus and Yang, 2015; Johnson and Whisman, 2013; Rassin and Muris, 2005; Piotrowski, 2019; Spunt et al., 2009) and the known association between socioeconomic status and depression (Freeman et al., 2016). Timepoint was added to help prevent confounds that may be related to seasonal or history effects. One model was fit for each sample. The two models were composed of 10 parameters including the intercept, the three main effects (abstract rumination, concrete rumination, aversive indecisiveness), the four covariates, and two interaction terms (abstract rumination by aversive indecisiveness and concrete rumination by aversive indecisiveness). Before running the model, all predictors were standardized (*M* = 0, *SD* = 1). We calculated Johnson-Neyman intervals to probe any significant interactions. Our figures depict simple slopes. For a formulaic representation of the model, please see the Supplementary material.

To assess multicollinearity, we calculated variance inflation factors (VIF). All VIFs were below the commonly used threshold of 5, except an interaction term and a constituent predictor in Sample 1, which had VIF's < 5.2, where moderate multicollinearity can be expected (Cohen et al., 2003, pp. 264, 425). To ensure that we obtained reliable estimates from our models, we tested our models to see if they met assumptions for ordinary least squares (OLS) regression. After running the regression analysis, visual inspection of the models' fitted values against their residuals demonstrated that there was heteroscedasticity in both models. As well, through creating normal probability plots of the models' residuals, we noticed that distribution of errors was right-skewed. To correct for both violations in OLS assumptions, a robust linear regression was used to re-run the models. This methodology is thought to provide robust estimates of the standard errors and down-weight outliers, thereby providing reliable significance tests of the model coefficients despite violated assumptions (Huber, 1973). The robust tests were run with an MM-estimator in R's *robustbase* package. From these, we provide pseudo *R*^2^ values. For robustness checks, we also ran the model using OLS regression with heteroskedasticity-consistent standard errors (i.e., HC3).

Analyses were carried out in R Statistical Software (v4.5.1; R Core Team, 2025). Specific packages that were used are as follows: *dplyr* for data cleaning (Wickham et al., 2023), *lavaan* for factor analysis (Rosseel, 2012), *psych* for scale reliability evaluation (Revelle, 2024), *Hmisc* for correlation matrix computation (Harrell Jr., 2025), *rstatix* for group comparisons (Kassambara, 2023), *robustbase* for robust linear modeling (Maechler et al., 2024), interactions for probing interactions (Long, 2024), broom for reporting regression results (Robinson et al., 2025), sandwich and lmtest for HC3 estimates (Zeileis et al., 2020; Zeileis, 2004; Zeileis and Hothorn, 2002), ggplot2 for plotting (Wickham, 2016), ggeffects for marginal effect predictions (for plotting; Lüdecke, 2018), patchwork for plot composition (Pedersen, 2025), and *report* for package citations (Makowski et al., 2023).

## 3 Results

### 3.1 Sample 1

#### 3.1.1 Descriptive statistics

On average, participants reported elevated levels of depressive symptoms (*M* = 12.7, *SD* = 10.7, range = 0-52.5). Based on cut scores on the BDI-II, ratings typically associated with non-clinical levels of symptoms were reported by 62.4% of participants, a mild level by 15.8%, a moderate level by 13.3%, and a severe level by 8.5%.

Correlations for all variables are displayed in Table 2. Of note, depressive symptoms, abstract rumination, and indecisiveness were all strongly positively correlated with one another. Concrete rumination was negatively correlated with these same variables. Regarding gender differences, women endorsed higher levels of depressive symptoms, *t*(244.63) = 4.55, *p* < 0.001, *d* = 0.48, higher levels of abstract rumination, *t*(215.82) = 4.26, *p* < 0.001, *d* = 0.46, and higher levels of aversive indecisiveness, *t*(203.99) = 5.19, *p* < 0.001, *d* = 0.57, than men. They also reported lower levels of concrete rumination, *t*(191.23) = − 2.99, *p* = 0.003, *d* = −0.33.

**Table 2:** Correlations for Sample 1.

	**Depressive symptoms**	**Abstract rumination**	**Concrete rumination**	**Aversive indecisiveness**	**Age**
1. Depressive symptoms	-				
2. Abstract rumination	0.67^***^	-			
3. Concrete rumination	−0.25^***^	−0.13^*^	-		
4. Aversive indecisiveness	0.49^***^	0.52^***^	−0.28^***^	-	
5. Age	0.07	−0.01	−0.02	−0.04	-
6. Income	−0.13^*^	−0.05	0.08	−0.15^*^	−0.10

^*^p < 0.05 ^***^p < 0.001.

#### 3.1.2 Moderating effect of indecisiveness on rumination and depressive symptoms

**Figure 1:**
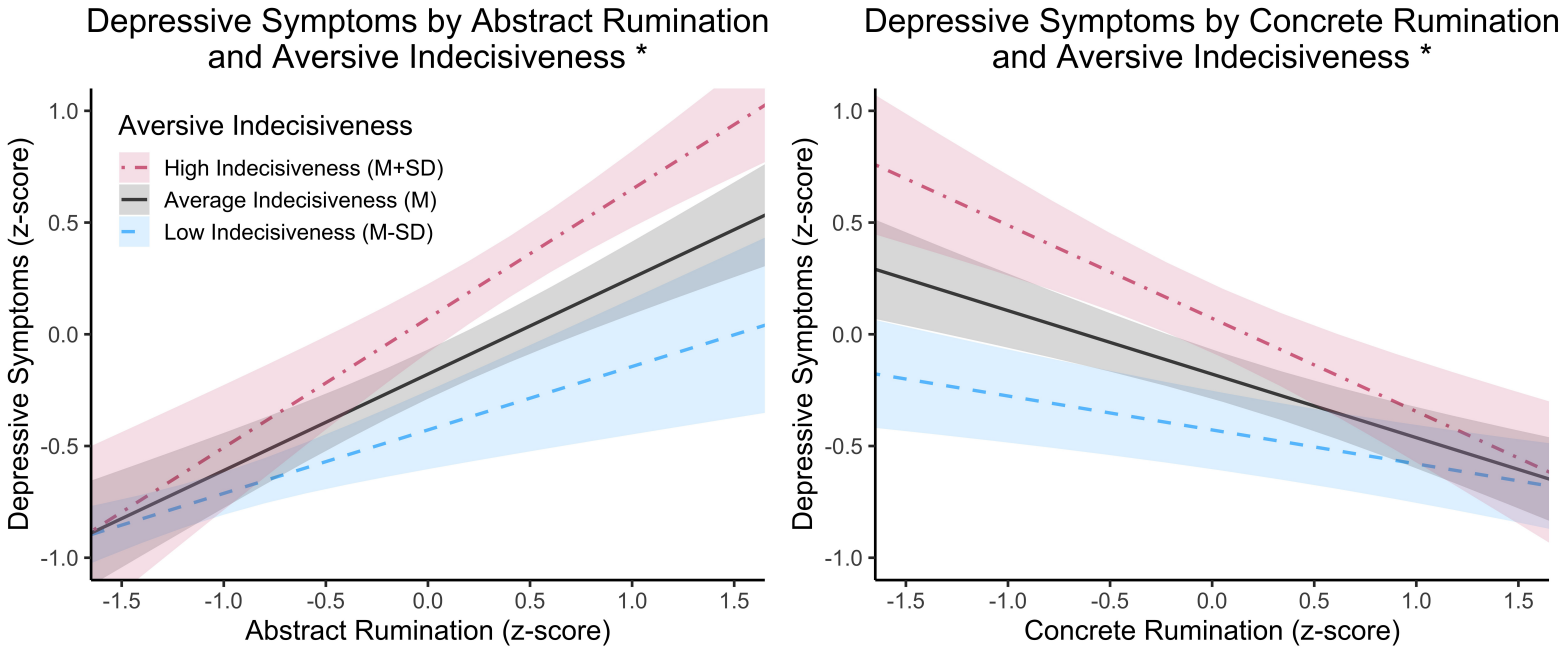
This graph depicts the interaction results from the linear regression model. On the y-axis is the predicted standardized score for an individual's reported depressive symptoms. On the x-axis is one's level of rumination, either abstract or concrete. The three lines on each plot depict one's levels of indecisiveness. Levels of low and high rumination and indecisiveness were values of M – SD and M + SD, respectively, based on our Sample 1 data. The predicted values were obtained assuming all variables not plotted were at a mean level. An asterisk (^*^) in the graph's title suggests the interaction between the predictors was significant at p < 0.05.

Utilizing a robust Wald test, the overall model fit was highly significant, *R*^2^ = 0.54, χ^2^(9) = 268.46, *p* < 0.001. The main effects of abstract rumination, concrete rumination, and aversive indecisiveness were all significant at *p* < 0.01. Regarding covariates, age, gender, timepoint, and income were not significant (all *p*'s >0.05). The interaction between abstract rumination and aversive indecisiveness was significant, *t*(403) = 2.07, *p* = 0.04. Specifically, the effect of abstract rumination on symptoms was significant when aversive indecisiveness was outside the interval −429 ≤ z ≤ −2.00 (where observed values of aversive indecisiveness for Sample 1 fell between −2.86 ≤ z ≤ 2.01). This interaction indicated that the positive relationship between abstract rumination and depressive symptoms becomes stronger as one's level of indecisiveness increases. The interaction between concrete rumination and aversive indecisiveness was not significant *t*(403) = − 0.66, *p* = 0.51. Plots depicting these interactions are visualized in Figure 1; plots for the Johnson-Neyman intervals are provided in the Supplementary material. Regression outputs for Sample 1 are provided in Table 3.

**Table 3:** Regression coefficients for sample 1.

**Predictor**	**B**	***SE***	***95% CI***	***p***
Intercept	10.77	0.46	[9.86, 11.67]	< 0.001
Abstract rumination	5.38	0.62	[4.18, 6.59]	< 0.001
Concrete rumination	−1.41	0.47	[−2.34,−0.48]	0.003
Aversive indecisiveness	1.81	0.65	[0.54, 3.09]	0.006
Abstract^*^Aversive indecisiveness	1.36	0.66	[0.07, 2.65]	0.04
Concrete^*^Aversive indecisiveness	−0.33	0.50	[−1.32, 0.66]	0.51
Gender	−0.65	0.67	[−1.96, 0.67]	0.33
Timepoint	−1.29	0.67	[−2.61, 0.03]	0.06
Age	0.32	0.54	[−0.74, 1.37]	0.56
Income	−0.39	0.38	[−1.14, 0.36]	0.30

*These results are from the regression model ran on Sample 2. Robust regression methodology was utilized, meaning that the estimates and standard errors have been adjusted due to detected heteroscedasticity. The outcome variable is depressive symptoms. All continuous predictors were standardized (M* = *0, SD* = *1). Gender was contrast coded such that woman* = −*0.5, man* = *0.5. Timepoint was contrast coded such that the sample from August-September 2024* = −*0.5, January-March 2025* = *0.5. B* = *estimates from model in terms of unstandardized outcome (score on BDI-II)*.

Regarding the robustness of results with different models, the direction and significance of estimates held when running the model using OLS regression and HC3 standard errors.

### 3.2 Sample 2

#### 3.2.1 Descriptive statistics

On average, participants reported elevated levels of depressive symptoms (*M* = 16.4, *SD* = 13.5, range = 0-57.8). The average score fell in the clinical range for mild depression on the BDI-II (a score of 13-19). Ratings typically associated with non-clinical levels of symptoms were reported by 49.2% of participants, a mild level by 15.5%, a moderate level by 17.8%, and a severe level by 17.4%.

Correlations for all variables are displayed in Table 4. Of note, depressive symptoms, abstract rumination, and indecisiveness were all strongly positively correlated with one another. Concrete rumination was negatively associated with these same variables. Regarding gender differences, women reported significantly higher levels of depressive symptoms *t* (243.98) = 2.05, *p* = *0.0*4, *d* = *0.2*6 than men. They also reported significantly lower levels of concrete rumination than men, *t* (244.55) = −2.57, *p* = 0.01, *d* = −0.33. There were no significant differences between women and men with respect to levels of abstract rumination (*p* = 0.78) or aversive indecisiveness (*p* = 0.054).

**Table 4:** Correlations for sample 2.

	**Depressive symptoms**	**Abstract rumination**	**Concrete rumination**	**Aversive indecisiveness**	**Age**
1. Depressive symptoms	-				
2. Abstract rumination	0.63^***^	-			
3. Concrete rumination	−0.38^***^	−0.12	-		
4. Aversive indecisiveness	0.59^***^	0.66^***^	−0.27^***^	-	
5. Age	−0.10	−0.23^***^	0.03	−0.20^**^	-
6. Income	−0.18^**^	−0.08	−0.05	−0.13^*^	0.05

^*^p < 0.05 ^**^ *p* < 0.01 ^***^p < 0.001.

#### 3.2.2 Moderating effect of indecisiveness on rumination and depressive symptoms

**Figure 2:**
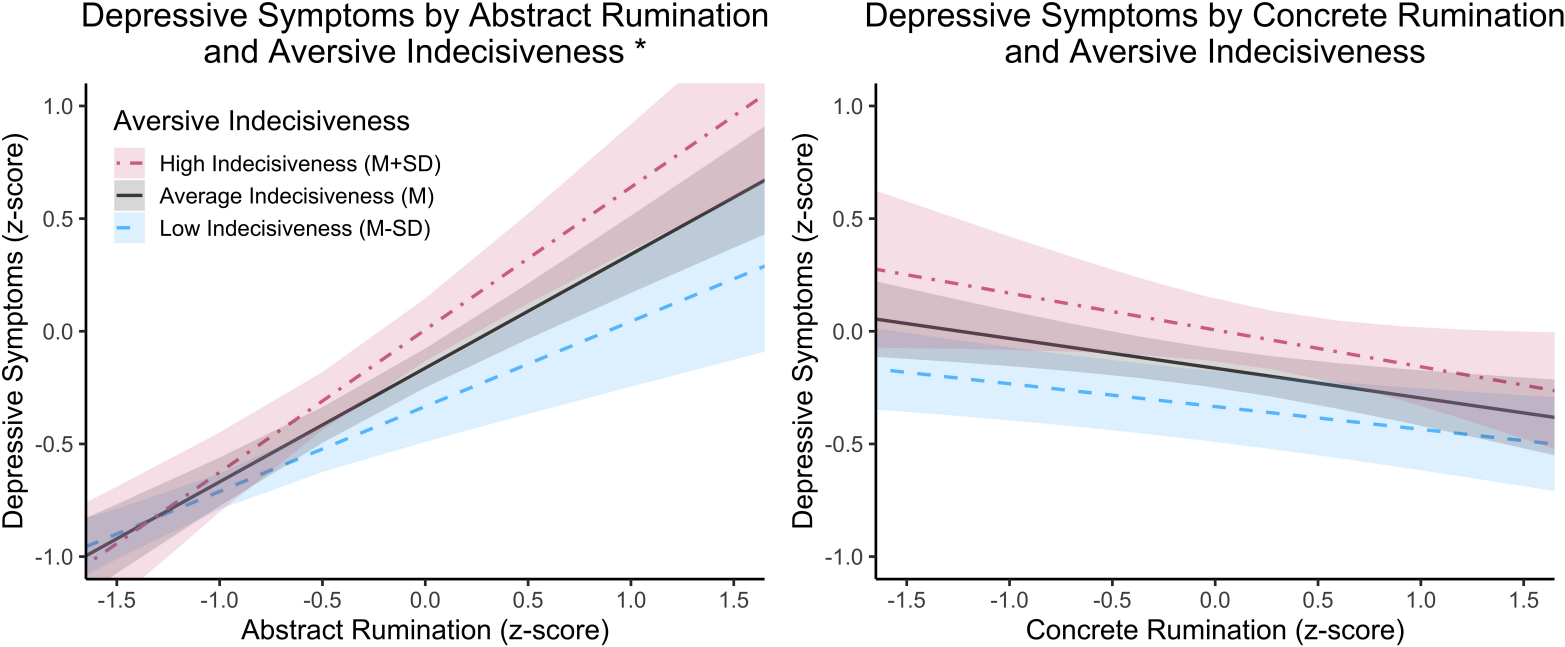
This graph depicts the interaction results from the linear regression model. On the y-axis is the predicted standardized score for an individual's reported depressive symptoms. On the x-axis is one's level of rumination, either abstract or concrete. The three lines on each plot depict one's levels of indecisiveness. Levels of low and high rumination and indecisiveness were values of M – SD and M + SD, respectively, based on our Sample 2 data. The predicted values were obtained assuming all variables not plotted were at a mean level. An asterisk (^*^) in the graph's title suggests the interaction between the predictors was significant at p < 0.05.

Utilizing a robust Wald test, the overall model fit was highly significant, *R*^2^ = 0.59, χ^2^ (9) = 391.57, *p* < 0.001. The main effects of abstract rumination, concrete rumination, and aversive indecisiveness were all significant at *p* < 0.001. Regarding covariates, income (*p* = 0.001) was significant but age, gender, and timepoint were not significant (all *p*'s >0.08). The interaction between abstract rumination and aversive indecisiveness was significant, *t*(248) = 2.93, *p* = 0.004. Specifically, the effect of abstract rumination on symptoms was significant when aversive indecisiveness was outside the interval −9.21 ≤ z ≤ −1.65 (where observed scores for aversive indecisiveness for Sample 2 fell between−2.22 ≤ z ≤ 1.83). This interaction between abstract rumination and aversive indecisiveness indicates that the positive relationship between abstract rumination and depressive symptoms becomes stronger as one's level of indecisiveness increases. In addition, the interaction between concrete rumination and aversive indecisiveness was also significant, *t*(248) = −3.42, *p* < 0.001. Specifically, the effect of concrete rumination on symptoms was significant when aversive indecisiveness was outside the interval −4.25 ≤ z ≤ −1.45. This interaction between concrete rumination and aversive indecisiveness indicates that the negative relationship between concrete rumination and depressive symptoms becomes stronger (more negative) as one's level of indecisiveness increases. Plots depicting these interactions are visualized in Figure 2; plots for the Johnson-Neyman intervals are provided in the Supplementary material. Regression outputs for Sample 2 are provided in Table 5.

**Table 5:** Regression coefficients for sample 2.

**Predictor**	**B**	***SE***	***95% CI***	***p***
Intercept	13.86	0.82	[12.26, 15.47]	< 0.001
Abstract rumination	5.81	0.84	[4.16, 7.47]	< 0.001
Concrete rumination	−3.83	0.72	[−5.24, −2.43]	< 0.001
Indecisiveness	3.37	0.84	[1.72, 5.01]	< 0.001
Abstract^*^Aversive indecisiveness	1.98	0.68	[0.66, 3.31]	0.004
Concrete^*^Aversive indecisiveness	−1.79	0.52	[−2.81, −0.76]	0.001
Gender	−0.57	1.09	[−2.71, 1.58]	0.61
Timepoint	0.50	1.26	[−1.96, 2.96]	0.69
Age	0.87	0.50	[−0.11, 1.84]	0.08
Income	−1.67	0.52	[−2.68, −0.65]	0.001

These results are from the regression model ran on Sample 2. Robust regression methodology was utilized, meaning that the estimates and standard errors have been adjusted due to detected heteroscedasticity. The outcome variable is depressive symptoms. All continuous predictors were standardized (M = 0, SD = 1). Gender was contrast coded such that woman = −0.5, man = 0.5, non-binary/non-response = 0. Timepoint was contrast coded such that the sample from August-September 2024 = −0.5, January-March 2025 = 0.5. B = estimates from model in terms of unstandardized outcome (score on BDI-II).

The direction and significance of results held when running the model using OLS regression and HC3 standard errors.

### 3.3 *Post-hoc* cross-sample model

Given minor discrepancies between the two samples' results, another model was run to test whether the interaction results significantly differed between samples. Utilizing the same model structure as specified in the Data Analysis Overview, we fit a robust linear model with data from both samples. The only changes were the addition of a sample covariate, sample-specific timepoint covariates, and sample by rumination by aversive indecisiveness interactions (i.e., one for abstract rumination, one for concrete rumination).

Utilizing a robust Wald test, the overall model fit was significant, *R*^2^ = 0.57, χ^2^ (13) = 568.9, *p* < 0.001. The main effects of abstract rumination, concrete rumination, and aversive indecisiveness remained significant at *p* < 0.001. Regarding covariates, sample (*p* = 0.012) and income (*p* = 0.015) were significant but age, gender, and timepoint were not significant (all *p*'s > 0.08). The interaction between abstract rumination and aversive indecisiveness was significant, *t*(657) = 4.35, *p* = 0.004, and so was the interaction between concrete rumination and aversive indecisiveness, *t*(657) = − 2.95, *p* = 0.003. These interactions were in the same directions as for the sample-specific models. Neither three-way interaction was significant (*p*'s > 0.58). The direction and significance of results held when running the model using OLS regression and HC3 standard errors.

## 4 Discussion

This was the first study to examine the moderating effect of aversive indecisiveness on the relationship between rumination modes and depressive symptoms. Overall, the findings from this study largely supported both our hypotheses and previous literature. Consistent with prior research, we replicated robust associations among rumination, aversive indecisiveness, and depressive symptoms. We found that abstract rumination was positively associated with both aversive indecisiveness and depressive symptoms. In contrast, we found that concrete rumination was negatively associated with indecisiveness and depressive symptoms. These patterns lend support to Watkins (2008) level of construal dysregulation theory, which proposes that the degree of abstraction in repetitive negative thinking is an important factor in its maladaptive consequences. Although the current study was correlational and cannot justify causal interpretations, the results are in line with previous results highlighting abstract repetitive negative thought as a particularly maladaptive mode of thinking (e.g., Di Schiena et al., 2013; Ros et al., 2024).

Supporting our first hypothesis, aversive indecisiveness significantly moderated the relationship between abstract rumination and depressive symptoms in both samples. Specifically, individuals high in both abstract rumination and aversive indecisiveness reported especially elevated depressive symptoms. In essence, when both cognitive vulnerabilities co-occur, their impact appears to be more than additive. This was replicated across the two independent samples. These findings strengthen the case for considering trait-level indecisiveness as an important factor in how rumination contributes to depression. Although previous work has demonstrated that induced abstract rumination can increase momentary indecision (van Randenborgh et al., 2010; Di Schiena et al., 2013), the present study extends this work by showing that stable patterns of abstract thinking and indecisiveness may jointly predict depressive symptom levels. This fits well within the framework identified in Watkins and Roberts (2020), which details how several factors, including level of abstraction, impaired executive function, and perceived goal discrepancies can interact in predicting the association between rumination and psychopathology risk.

These findings have meaningful implications for intervention. Rumination-focused cognitive behavioral therapy (RFCBT; see Watkins, 2018 for a training manual) has already shown promise in reducing depressive symptoms by targeting rumination. Our results suggest that such treatments could be enhanced by addressing indecisiveness as a co-occurring risk factor. Interventions that target decision aversion, alongside attempting to reduce abstract repetitive thinking, may offer greater efficacy especially among individuals presenting with deficits in both.

Regarding our second hypothesis, findings for the moderating effect of aversive indecisiveness on the relationship between concrete rumination and depressive symptoms were less consistent. In the general population sample, we observed a significant interaction such that the negative association between concrete rumination and depressive symptoms was stronger at higher levels of indecisiveness. This was contrary to our prediction that indecisiveness would weaken this protective association. While this effect was not significant in the undergraduate sample, both regression coefficients and visual inspection of the plotted interaction effects indicate that the direction of the interaction was the same in both samples (see Figures 1, 2). One potential explanation for the null result is that students may not have reported depressive symptoms at a level high enough for a buffering effect of concrete rumination to be found (i.e., a floor effect). However, it is also possible that this interaction may not exist for students (i.e., concrete rumination may not be differentially protective depending on level of indecisiveness for students). A *post-hoc* analysis with both samples supported the two-way interaction between concrete rumination and aversive indecisiveness, and this interaction was not found to differ across sample (i.e., the three-way interaction was not significant). It is possible that this test was underpowered, and the interaction between concrete rumination and aversive indecisiveness should still be interpreted cautiously. However, these preliminary findings provide reason for doing further work in this area. For instance, work adjacent to RFCBT has looked at the impact of concreteness training, an intervention designed to increase concrete rumination (Watkins and Moulds, 2005; Watkins et al., 2009). Our results raise the possibility that such training may be especially effective for individuals who also exhibit indecisiveness. Future work may benefit from examining whether indecisiveness moderates treatment response to RFCBT or concreteness training, particularly in how gains in concrete rumination may contribute to symptom reduction.

Lastly, this study provides psychometric support for several measurement instruments. This was the first known study using the Mini-CERTS in English. In the present study, we identified two factors aligned with theoretically supposed dimensions using exploratory factor analysis. We also identified good and adequate internal reliability for the abstract, concrete subscales of the Mini-CERTS, providing preliminary support for its use in future work. Further, this study replicated the factor structure of the Revised Indecisiveness Scale (Rassin et al., 2007) identified in Lauderdale and Oakes (2021). Given idiosyncratic approaches used in past indecisiveness research, this is a step toward a unified view of the construct of indecisiveness. Particularly, we replicated the important association between the aversive indecisiveness subscale and psychopathology risk (i.e., concurrent depressive symptoms).

### 4.1 Limitations

There are several limitations of this study. First, the study was cross-sectional and correlational, limiting our ability to draw causal inferences. Longitudinal and experimental designs would be better suited to examine the temporal dynamics and causal relationships between rumination, indecisiveness, and depressive symptoms. Second, although participants in both samples endorsed heightened levels of depressive symptoms, neither sample was recruited as a clinical sample. Replication studies and future work should be done to confirm how these results may generalize to participants with MDD. Third, while the Mini-CERTS provided a theoretically motivated measure of rumination modes, this is first study to utilize this scale in English. With this, we identified some potential issues with the factor structure and measurement invariance. Overall, given the exploratory nature of this study, our factor and reliability analyses supported its use, but further psychometric work should be done to refine the Mini-CERTS. Similarly, although a previous investigation guided our measure of aversive indecisiveness, further validation work would provide more confidence in the aversive indecisiveness subscale. Lastly, both the indecisiveness and rumination scales contain emotionally laden terms. This means there is a possibility that estimated associations between these variables and our measure of depressive symptoms were over-inflated given theoretical overlap.

### 4.2 Conclusions

This study contributes to the growing literature linking rumination modes and indecisiveness to depressive symptoms, providing new evidence that their interaction may be particularly important. Specifically, abstract rumination and aversive indecisiveness were synergistic trait-level factors associated with heightened depressive symptoms. Conversely, concrete rumination may serve a protective role in certain circumstances, particularly among individuals with higher levels of indecisiveness. We suggest future research investigating the causal relationships between rumination and indecisiveness in individuals with clinical depression. Such approaches could help us to better understand how to tailor clinical intervention to address these risk factors, thereby helping us to improve our treatment of depression.

## Data Availability

The raw data supporting the conclusions of this article will be made available by the authors, without undue reservation.

## Footnotes

1 We conducted a formal multi-group confirmatory factor analysis to assess measurement invariance. The results indicated poor fit (CFI = 0.80; TLI = 0.76; RMSEA = 0.098; SRMR = 0.097). Therefore, it is unclear whether the factor structure is invariant. Given theory-consistent results from the EFA, we continued with calculating sub scores in the current study but primarily analyzed data separately for each sample.
