# Supplementary material for: Indecisiveness moderates the relationship between rumination modes and depressive symptoms
Source: Front Psychol. 2025 Nov 6;16:1681121. doi: 10.3389/fpsyg.2025.1681121 (PMC12631396; doi:10.3389/fpsyg.2025.1681121)
Supplement: Supplementary file 1 [file Table_1.docx]

Supplementary Material

# Comparisons Between Timepoints Within Each Sample

**Table A1**

*Sample 1 (Undergraduate Students) Descriptive Statistics Between Timepoints*

|  | Timepoint 1 (*n* = 244) | | Timepoint 2 (*n* = 168) | |
| --- | --- | --- | --- | --- |
| Variables | Mean | SD | Mean | SD |
| Depressive symptoms | 13.25 | 10.62 | 11.99 | 10.69 |
| Abstract rumination | 23.35 | 5.01 | 22.98 | 4.80 |
| Concrete rumination | 15.11 | 3.15 | 15.21 | 3.06 |
| Indecisiveness | 13.38 | 3.34 | 13.47 | 3.20 |

*Note*. Timepoint 1 was collected from August-September 2024. Timepoint 2 was collected in January-March 2025.

**Table A2**

*Sample 2 (Adults from General US population) Descriptive Statistics Between Timepoints*

|  | Timepoint 1 (*n* = 72) | | Timepoint 2 (*n* = 186) | |
| --- | --- | --- | --- | --- |
| Variables | Mean | SD | Mean | SD |
| Depressive symptoms | 16.39 | 13.41 | 15.29 | 12.64 |
| Abstract rumination | 23.15 | 5.62 | 21.67 | 5.69 |
| Concrete rumination | 14.93 | 3.87 | 14.76 | 3.45 |
| Indecisiveness | 13.22 | 4.09 | 12.61 | 3.89 |
| Age | 35.67 | 12.96 | 35.59 | 11.97 |

*Note*. Timepoint 1 was collected in July-August 2024. Timepoint 2 was collected in February 2025.

# Factor Analyses

## Mini-CERTS Factor Analysis

Exploratory factor analysis (EFA) was performed to evaluate the fit of the Mini-CERTS data. EFA was used because no previous studies have dealt with the Mini-CERTS in English. The structures obtained for both samples were compared to the subscales initially conceived by Douilliez et al. (2014).

In Sample 1 (undergraduate students), the data demonstrated adequate sampling adequacy through the Kaiser-Meyer-Olkin test, KMO = .82, and sufficient factorability based on the Bartlett sphericity tests, $\chi^{2}$(120) = 1738.03, *p* < .001. Both theoretical underpinnings and previous empirical work suggested that the EFA should be specified with two factors. This was supported by the inspection of the parallel analysis scree plot, with two observed eigenvalues being greater than those tied to simulated data (3.92, 2.83; using principal axis factoring looking at principal components; fa.parallel() in psych package in R [Revelle, 2024]). With two factors, the EFA model explained 34.29% of the variance. Adding a third factor only increased the variance explained by an additional 4.66%. After an oblique rotation (i.e., using oblimin), all items but one loaded only on their respective factors as theorized (see Douilliez, et al., 2014) with loadings of λ ≥ .3 (see Table B1 for all factor loadings ). Item 16, which was expected to load on the concrete rumination factor, had an equally large cross-loading on the abstract rumination factor. Therefore, it was the only item removed before sum scores were calculated for each subscale. The sum score for abstract rumination showed good internal consistency (ɑ = .81, 95% CI [78, .84]) and the sum score for concrete rumination showed acceptable internal consistency (ɑ = .71, 95% CI [.67, .76]).

In Sample 2 (general population adults), the data also demonstrated adequate sampling adequacy through the Kaiser-Meyer-Olkin test, KMO = .85, and sufficient factorability based on the Bartlett sphericity tests, $\chi^{2}$(120) = 1407.73, *p* < .001. As with Sample 1, this EFA was specified with two factors. This choice was supported by the inspection of the parallel analysis scree plot, with two observed eigenvalues being greater than those tied to simulated data (4.48, 3.12; using principal axis factoring looking at principal components; fa.parallel() in psych package in R [Revelle, 2024]). With two factors, the EFA model explained 40.51% of the variance. Adding a third factor only increased the variance explained by an additional 4.3%. After an oblique rotation (using oblimin), all items loaded on their respective factor as theorized (see Douilliez, et al., 2014) with loadings of λ ≥ .3 (see Table B1 for all factor loadings). Therefore, all items were retained in calculating subscale sum scores. The sum score for abstract rumination showed good internal consistency (ɑ = .85, 95% CI [.83, .88]) and the sum score for concrete rumination acceptable internal consistency (ɑ = .79, 95% CI [.74, .83]).

**Table B1**

*Factor Loadings by Item and Sample for Mini-CERTS*

|  | Sample 1 | | Sample 2 | |
| --- | --- | --- | --- | --- |
| Item No. | Factor 1 | Factor 2 | Factor 1 | Factor 2 |
| Item 1 | .52 |  | .65 |  |
| Item 2 |  | .41 | -.35 | .39^b^ |
| Item 3 | .68 |  | .65 |  |
| Item 4 |  | .64 |  | .67 |
| Item 5 | .46 |  | .39 |  |
| Item 6 | .44 |  | .57 |  |
| Item 7 | .58 |  | .70 |  |
| Item 8 |  | .62 |  | .69 |
| Item 9 |  | .57 |  | .54 |
| Item 10 | .55 |  | .64 |  |
| Item 11 |  | .34 |  | .56 |
| Item 12 | .63 |  | .74 |  |
| Item 13 |  | .59 |  | .80 |
| Item 14 | .58 |  | .64 |  |
| Item 15 | .69 |  | .67 |  |
| Item 16 | .35^a^ | .42^a^ |  | .31 |

*Note*. These results were from an exploratory factor analysis and each of these was conducted with only the data from one sample. Factor loadings with magnitude | λ | > .30 are included in the table. Factor 1 is aligned with the theoretical definition of abstract/unconstructive rumination and Factor 2 is aligned with the theoretical definition of concrete/constructive rumination.

^a^ Given similarly large loadings in both directions, this item was not used in the calculation of either sum score for Sample 1.

^b^ This item was determined to *primarily load* on factor 2 in Sample 2 given theoretical motivation (and use of only *positive loadings* in all other cases). Therefore, it was used for the concrete rumination sum score in Sample 2.

**Table B2**

*Factor Loadings by Item and Sample for the Revised Indecisiveness Scale*

| Factor Loadings | | | | | |
| --- | --- | --- | --- | --- | --- |
| Item | Sample 1 | | Sample 2 | | |
|  | Positive | Aversive | Positive | | Aversive |
| RIS2 | .67 |  | .75 | |  |
| RIS3 | .89 |  | .90 | |  |
| RIS4 | .64 |  | .71 | |  |
| RIS5 | .64 |  | .66 | |  |
| RIS6 | .75 |  | .77 | |  |
| RIS7 | .47 |  | .65 | |  |
| RIS8 |  | .79 |  | | .88 |
| RIS9 |  | .75 |  | | .80 |
| RIS10 |  | .67 |  | | .74 |
| RIS11 |  | .66 |  | | .72 |
| Modeled Item Covariances | | | | | |
| Item 1 | Item 2 | Sample 1 Estimate | | Sample 2 Estimate | |
| RIS2 | RIS9 | .02 ^ns^ | | -.02 ^ns^ | |
| RIS3 | RIS9 | .11 ^ns^ | | .21 | |
| RIS4 | RIS5 | .15 | | .13 | |
| RIS5 | RIS7 | .19 | | .25 | |
| RIS5 | RIS10 | -.39 | | -.33 | |
| RIS6 | RIS11 | .03 ^ns^ | | -.13 ^ns^ | |

*Note*. This above table gives factor loadings and modeled item (i.e., indicator) covariances for a confirmatory factor analysis with the specification described in Lauderdale and Oakes (2021). Loadings and covariances provided are the standardized estimates, that is, with both the observed scores and latent variables standardized (i.e., variance fixed to 1). Only the items specified to load onto the aversive indecisiveness factor were used to make a sum score for further analyses in this study, but the full factor structure is included for the sake of completeness. RIS = Revised Indecisiveness Scale (Rassin et al., 2007). Positive = Positive Attitudes Towards Decision-Making factor. Aversive = Aversive Indecisiveness factor.

^ns^ = not significant at p < .05 (i.e., all other loadings/covariances were significant)

# Model Equation

Provided below is a mathematical depiction of the regression equation specified for our primary analyses:

$$\hat{\mathrm{DeprS}x_{i}}=b_{0}+b_{1}\mathrm{Abstrac}t_{i}+b_{2}\mathrm{Concret}e_{i}+b_{3}\mathrm{In}d_{i}+b_{4}\mathrm{Abstrac}t_{i}\mathrm{Concret}e_{i}+b_{5}\mathrm{Abstrac}t_{i}\mathrm{In}d_{i}+b_{6}\mathrm{Gende}r_{i}+b_{7}\mathrm{Timepoint}_{i}+b_{8}\mathrm{Age}_{i}+b_{9}\mathrm{Income}_{i}$$

where,

- $\hat{\mathrm{DeprS}x_{i}}$ reflects the model-estimated value for an individual’s depressive symptoms,
- $b_{i}$ reflects an estimate of a model parameter/coefficient,
- $\mathrm{Abstrac}t_{i}$ reflects a standardized measure of an individual’s level of abstract rumination,
- $\mathrm{Concret}e_{i}$ reflects a standardized measure of an individual’s level of concrete rumination,
- $\mathrm{In}d_{i}$ reflects a standardized measure of an individual’s level of indecisiveness,
- $\mathrm{Gende}r_{i}$ is a contrasted-coded variable representing an individual’s gender
  - Coding was as follows: men = 0.5, women = -0.5, nonbinary/other/prefer not to answer = 0
- $\mathrm{Timepoint}_{i}$ is a contrast-coded variable representing an individual’s timepoint of measurement
  - Coding was as follows:
    - Sample 1: August-September 2024 = -0.5, January-March 2025 = 0.5
    - Sample 2: July-August 2024 = -0.5, February 2025 = 0.5
- $\mathrm{Age}_{i}$ is a standardized measure of an individual’s age
- $\mathrm{Income}_{i}$ is a standardized measure of an individual’s household income
  - Measured originally as a multiple-choice question with income intervals in dollars per annum and then converted into a continuous variable by using interval midpoints
  - Missing data was imputed using single imputation of the mean per each sample

# Johnson-Neyman Plots

**Figure D1**

*Johnson-Neyman Plots for Sample 1*

**
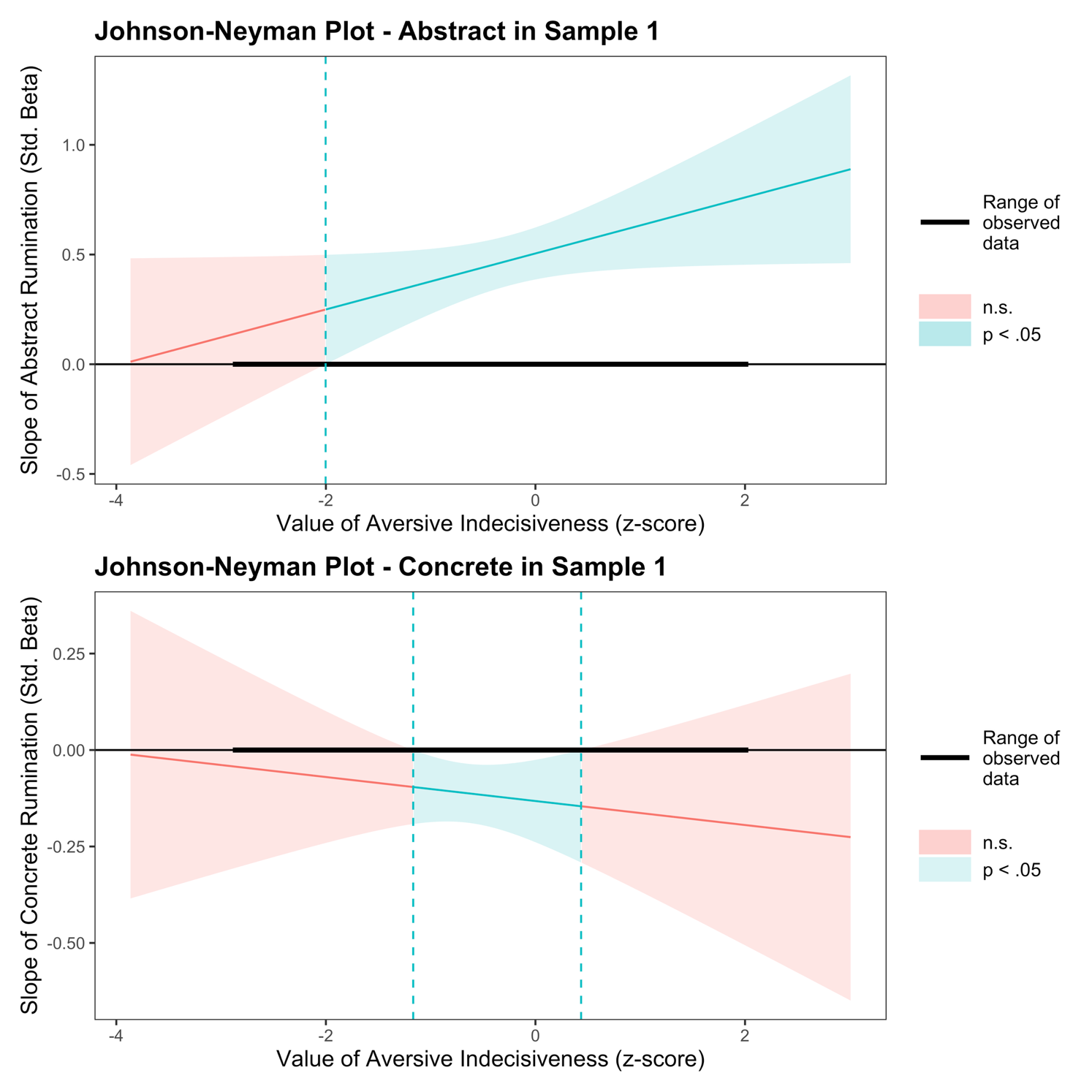
**

*Note.* The interaction between concrete rumination and aversive indecisiveness was not significant for this sample.

**Figure D2**

*Johnson-Neyman Plots for Sample 2*

*
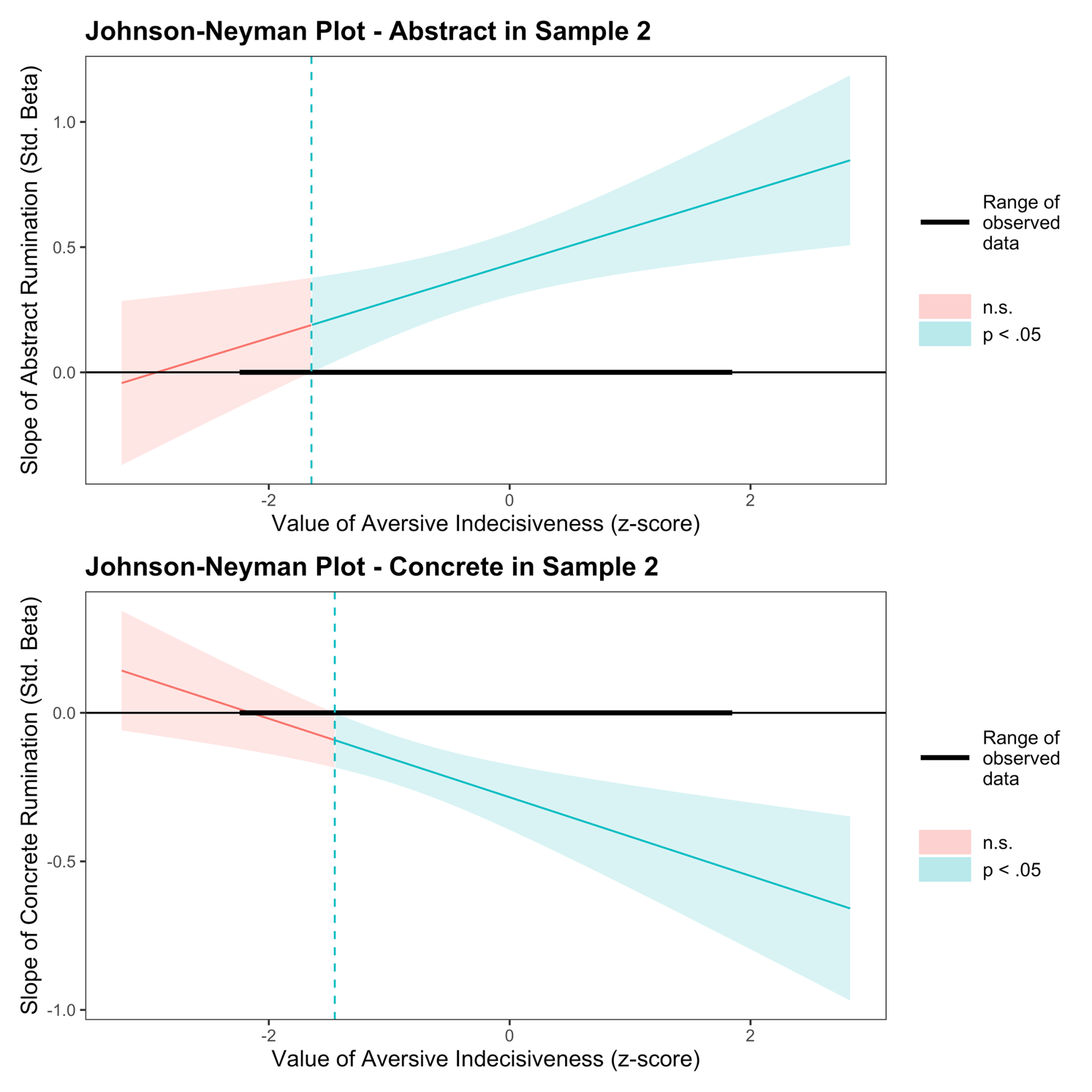
*
